# Risk Determinants of Dental Caries and Oral Hygiene Status in 3–15 Year-Old Recent Immigrant and Refugee Children in Saskatchewan, Canada: A Pilot Study

**DOI:** 10.1007/s10903-016-0452-9

**Published:** 2016-06-27

**Authors:** Jay Hoover, Hassan Vatanparast, Gerry Uswak

**Affiliations:** 1 College of Dentistry, University of Saskatchewan, 105 Wiggins Road, Saskatoon, SK Canada; 2 College of Pharmacy and Nutrition, University of Saskatchewan, Saskatoon, SK Canada

**Keywords:** Oral health, New immigrants, Refugees, Saskatchewan

## Abstract

This study aimed to identify the risk determinants of caries and record oral hygiene status in recent immigrant and refugee children residing in Saskatoon and Regina, Saskatchewan, Canada. Convenience samples of 133, 3–15 year-old recent immigrant and refugee children, and 86 adult guardians were recruited. Clinical examination of children and survey of their guardians explored the presence of at least one decayed tooth in the child’s mouth; and the knowledge, attitudes, behaviors, among other aspects in adult participants. Refugee children had statistically significant higher decayed, missing, filled teeth (DMFT) scores (mean dmft/DMFT score 5.80 ± 4.24) than immigrant children (mean dmft/DMFT score 3.52 ± 3.78 (*p* < 0.001). Adult immigrants had significantly higher proficiency in English language, knowledge about preventive components like fluoride and dental floss compared to refugee adults. The results of this study confirm the poorer state of oral health among refugee and immigrant children compared to Canadian children.

## Background

Immigration has had its presence and effects on Canada for a very long time, resulting in a significant number of immigrants and refugees living in this country today [1, 2]. Preventable oral diseases such as caries are expensive to manage, especially in Canada, affecting the economy through lost work time and school days, [3]. The pain and discomfort associated with unmet dental treatment needs could result in decreased school attendance and performance [4, 5].

Studies indicate that recent immigrant children and adults have higher unmet oral health needs and appear to be at a greater risk for oral and dental diseases compared to their native counterparts [5]. A Greek study reported that immigrant children had 1.68–4.34 higher odds for higher decayed, missing and filled teeth scores (DMFT values), higher unmet treatment needs and poorer oral hygiene levels than their Greek counterparts. Additionally, children from lower income areas were 1.2–2.14 times at greater risk for developing an increased caries severity and poorer oral hygiene [6]. A similar trend in oral health has been documented, primarily in Central Canada [7–12]. The reasons for this are varied and include a lack of finances and dental insurance, discrepancies in oral health knowledge, beliefs and attitudes, a lack of motivation and an underutilization of available dental service especially among low-income groups [13–15]. Over the recent years, two major cities in the province of Saskatchewan, Saskatoon, and Regina, have become home to a large number of new immigrant and refugees. However, published data on the oral health status, its risk determinants and treatment needs of this population group is scarce in Western Canada, including Saskatchewan. Such data is necessary to plan and implement relevant intervention strategies to improve the oral health of adults and children alike. This study was therefore undertaken to fill this crucial gap in the existing literature.

### Theoretical Framework

A multitude of enabling factors such as a lack of finances and dental insurance, discrepancies in oral health knowledge, beliefs and attitudes stress, depression, low utilization of health services, and lack of motivation make recent immigrants and refugees highly susceptible to the development of oral diseases [13–15].

## Methods

Convenience samples (due to budgetary and time constraints) of 133, 3–15 year-old recent immigrant and refugee children and 86 adult guardians were recruited in Saskatoon and Regina. Each participant had arrived in Canada within the last 7 years. The subjects were part of a larger study referred to as ‘Healthy Immigrant Children Research’ study, which assessed the general health and nutrition, socioeconomic and food security status in this sample. Informed consent was obtained from the adult guardians prior to the administration of the survey and initiation of the clinical examination. The data were collected from September 2012 to June 2013.

### Clinical Examination

The clinical examinations were conducted by two experienced clinicians (one in each locale) using a portable dental unit, dental mirror and a dental explorer. The permanent, deciduous and mixed dentitions were analyzed together. The caries status was assessed in terms of the presence of the number of decayed, missing and filled teeth (dmft/DMFT). The second molars were excluded as these teeth were fully erupted in only a few participants. Oral hygiene status (debris and calculus) was evaluated by means of the Simplified Oral Hygiene Index (OHIS) described by Green and Vermillion [16] on the following teeth: permanent maxillary right first molar, right central incisor, left the first molar, permanent mandibular left first molar, left central incisor, right first molar. Primary maxillary right central incisors, mandibular left central incisors, and first molars were assessed when applicable. The OHIS scores for each participant was calculated before comparing the scores between the two groups. Disclosing agents were not used. The absence of gingivitis upon visual inspection was recorded if the gingiva appeared to be clinically healthy and showed no signs of inflammation such as redness and swelling. The gingival status was recorded on the same teeth and surfaces used to score for debris. Probing of the soft tissues was not attempted and no radiographs were taken. Overall treatment needs for urgent treatment for pain and infection, extractions, restorations, orthodontics, plaque control instructions and scaling; root planning were also recorded.

### Questionnaire

All adults accompanying the children completed a questionnaire comprising of selected questions adapted from the literature [17–20] aiming to elicit oral health knowledge and practices, perceived oral health status and perceived barriers to oral care in Saskatchewan. The survey instrument facilitated a face-to-face interview with trained interpreters. The study was conducted in full accordance with ethical principles and with the approval of the Behavioral Research Ethics Board, University of Saskatchewan, Canada. The descriptive analysis was done using SPSS 20.0 and SAS 9.3 was used to perform regression analysis. Descriptive results were presented as means and standard deviations for continuous variables. For categorical variables, the distribution of participants across variables of interest was calculated. A comparison across immigrant and refugee groups was carried out using Independent *t* test and Mann–Whitney U tests, as appropriate for continuous variables and Chi square test and Fisher’s exact tests, as applicable for categorical variables. The outcome of interest for logistic regression was the presence or absence of at least one carious tooth in the child’s mouth. Alpha was set at the level of 0.05 in all analyses.

## Results

### Clinical Examination

The immigrant group consisted of 44 children/adolescents (22 females and 22 males), mean age; 8.63 ± 2.96 years (14.51–3.18 years); refugee group consisted of 89 participants (53 males and 36 females), mean age; 9.22 ± 2.89 years (range 15.17–3.18 years) (Table 1). The Oral Hygiene Index Scores (OHIS) for both the groups were comparable without any statistically significant difference; the mean OHIS scores were 1.51 ± 0.88 and 1.57 ± 0.99 for immigrant and refugee groups respectively (Table 2). Refugees had statistically significant higher scores (mean dmft/DMFT score 5.80 ± 4.24) than immigrants, (mean dmft/DMFT score was 3.52 ± 3.78 (*p* < 0.001) (Table 2) However, only the filled teeth scores differed significantly between the two groups, with a mean score of 0.48 ± 1.52 for the immigrants and 1.55 ± 2.36 for refugees (*p* < 0.001) (Table 3). Inflammation scores and the requirement for other treatment needs did not differ significantly approximately 70 % of our sample had visible signs of gingival inflammation. The majority of children required a restorative treatment, scaling and plaque control instructions (Table 4).

**Table 1:** Demographic characteristics of recent immigrants and refugee participant children

	Immigrants (*n* = 44)	Refugees (*n* = 89)	Total (*n* = 133)	*p* value
Sex distribution				
Males	22 (50 %)	53 (59.6 %)	75 (56.4 %)	
Females	22 (50 %)	36 (40.4 %)	58 (43.6 %)	
Age				
Mean	8.63 ± 2.96	9.22 ± 2.89	9.02 ± 2.91	0.28*
Maximum	14.51	15.17	15.17	
Minimum	3.18	3.22	3.18	
3–6 years	11 (25 %)	14 (15.7 %)	25 (18.8 %)	0.35**
>6–14 years old	32 (72.5 %)	70 (78.7 %)	102 (76.7 %)	
>14–16 years old	1 (2.3 %)	5 (5.6 %)	6 (4.5 %)	

* Independent samples *t* test

** Fisher’s exact test

**Table 2:** Oral Hygiene Index and DMFT Score in recent immigrants and refugee children

	Immigrants	Refugees	*p* value
OHIS scores (mean)	1.51 ± 0.88	1.57 ± 0.99	0.86*
Maximum	3.17	6	
Minimum	0	0	
DMFT score (mean)	3.52 ± 3.78	5.80 ± 4.24	
Maximum	12	16	
Minimum	0	0	
Median	2	5	<0.001*
DMFT score categories			
DMFT score = 0–3	26 (59.1 %)	32 (36 %)	0.04**
DMFT score = 4–8	11 (25 %)	32 (36 %)	
DMFT score ≥ 9	7 (15.9 %)	25 (28 %)	

Statistically significant values (*p* < 0.05) are given in bold

* Mann–Whitney U test

** Fisher’s exact test

**Table 3:** Decayed, missing, filled teeth in a group of recent immigrants and refugee children in Saskatchewan

	Immigrants (*n* = 44)	Refugees (*n* = 89)	*p* values
No. of decayed teeth			
Mean	2.41 ± 3.44	3.01 ± 3.49	
Maximum	12	13	
Minimum	0	0	
Median	1	2	0.22*
No. of Subjects with			
0–3 decayed teeth	33 (75 %)	60 (67.4 %)	0.74**
4–8 decayed teeth	7 (15.9 %)	19 (21.3 %)	
≥9 decayed teeth	4 (9.1 %)	10 (11.2 %)	
No. of missing teeth			
Mean	0.64 ± 1.12	1.25 ± 2.20	
Maximum	4	12	
Minimum	0	0	
Median	0	0	0.08*
No. of subjects with			
0–3 missing teeth	42 (95.5 %)	83 (93.3 %)	0.70**
4–8 missing teeth	2 (4.5 %)	3 (3.4 %)	
≥9 missing teeth	0 (0 %)	3 (3.4 %)	
No. of filled teeth			
Mean	0.48 ± 1.52	1.55 ± 2.36	
Maximum	8	9	
Minimum	0	0	
Median	0	0	<0.001*
No. of subjects with			
0–3 filled teeth	41 (93.2 %)	71 (79.8 %)	0.16**
4–8 filled teeth	3 (6.8 %)	16 (18 %)	
≥9 filled teeth	0 (0 %)	2 (2.2 %)	

Statistically significant value (*p* < 0.05) is given in bold

* Mann–Whitney U test

** Fisher’s exact test

**Table 4:** Other treatment needs in a group of recent immigrants and refugees in Saskatchewan

Need	Immigrants	Refugees	*p* value
Urgent treatment for pain and infection	3 (6.8 %)	4 (4.5 %)	0.68*
Extraction or surgery	3 (6.8 %)	10 (11.2 %)	0.54*
Restorations	24 (54.5 %)	51 (57.3 %)	0.76**
Plaque control instruction	42 (95.5 %)	81 (91 %)	0.50*
Scaling and root planing	20 (45.5 %)	41 (46.1 %)	0.95**
Orthodontic treatment	15 (34.1 %)	35 (39.3 %)	0.56**

* Fisher’s exact test

** Pearson’s Chi square test

#### Risk Determinants

Among the potential predictor variables analyzed, age of the parent, parents’ views about brushing teeth after meals, oral hygiene status of the child’s mouth, presence of inflammation in the child’s mouth and place of origin (for analysis, the countries were divided into three broad categories—Indian subcontinent, other parts of Asia, and the rest of the world) were found to be statistically significant in the univariate analysis (*p* value = 0.2).

Backward selection of the variables was used to build the model wherein, all the statistically significant predictor variables (as determined by univariate analysis) were included and modeled repeatedly while eliminating non-significant variables at *p* value 0.05 until all the variables included in the model were statistically significant. In multivariate analysis, country of origin, and gingival inflammation were found to be significant determinants for caries. The odds of the presence of at least one carious tooth in participants who came from other parts of Asia, excluding the Indian subcontinent (most of whom were refugees), were 3.54 (95 % CI 1.10–11.37) times more than participants from the category—rest of the world. Similarly, the odds of the presence of at least one carious tooth were 2.31 (95 % CI 0.94–5.66) times more in the presence of general inflammation (Table 5).

**Table 5:** Risk determinants in a group of recent immigrants and refugee children

Parameter	Estimate (adjusted model)	Odd’s ratio	95 % CI
Intercept	−1.56		
Origin	1.26	3.54	1.10–11.37
*Other Asia*			
General inflammation	0.84	2.31	0.94–5.66
Oral Hygiene Index	1.00	2.72	0.59–12.59

Results of logistic regression analyses

### Survey Questionnaire

The questionnaire (Table 6) was completed by 28 immigrant (22 females and 6 males) and 58 refugee (34 female and 24 male) parents/guardians of the children. The mean age of the immigrant participants was 38.07 ± 5.21 years (range 29–48 years), and the mean age of refugee participants was 36.14 ± 8.02 years (range 20–57 years). The majority of immigrants arrived from Pakistan and refugees came from Burma.

**Table 6:** Selected questions relating to self-reported knowledge, behavior and attitudes towards oral health

	Immigrant adults (*n* = 28)	Refugee adults (*n* = 58)	*p* value
Age of the participant			
Mean	38.07 ± 5.21	36.14 ± 8.02	0.09*
Minimum	29	20	
Maximum	48	57	
Sex of the participant			
Male	6 (21 %)	24 (41 %)	0.07**
Female	22 (79 %)	34 (59 %)	
Should you clean your teeth after meals?			
Yes	25 (89 %)	36 (62 %)	0.009**
No	3 (11 %)	22 (38 %)	
Are sweets most harmful for your teeth?			
Yes	26 (93 %)	54 (93 %)	1.00^†^
No	2 (7 %)	4 (7 %)	
What causes a tooth to decay?			**0.002** ^†^
Gave the correct answer	22 (79 %)	29 (50 %)	
Gave wrong answer	3 (11 %)	2 (3 %)	
Do not know	3 (11 %) Std res (-2.2)	27 (47 %)	
Have you heard of Dental Plaque?			**0.003** ^†^
Yes	18 (64 %) Std res = 2.1	16 (28 %)	
No	8 (29 %)	25 (43 %)	
Do not know	2 (7 %)	17 (29 %)	
What is dental floss?			0.25^†^
Correct explanation	17 (61 %)	26 (45 %)	
Wrong explanation	1 (4 %)	1 (2 %)	
Do not know	10 (36 %)	31 (53 %)	
Does fluoride make your teeth strong?			**<0.001** ^†^
Yes	21 (75 % of 28) Std res = 2.7	15 (26 % of 58)	
No	1 (4 % of 28)	4 (7 % of 58)	
Barriers			
Lack of money	14 (50 %)	32 (55 %)	0.76^†^
Lack of insurance	14 (50 %)	24 (41 %)	0.76^†^
Fear of pain	5 (18 %)	14 (24 %)	0.72^†^
English language	2 (7 %)	33 (57 %)	**<0.0001** ^†^
Work schedule conflict	4 (14 %)	11 (19 %)	0.84^†^
Lack of transportation	3 (11 %)	13 (22 %)	0.41^†^

Statistically significant values (*p* < 0.05) are given in bold

* Independent samples *t* test

** Pearson’s Chi square test

^†^Fisher’s exact test

A significantly higher number of immigrant participants were aware of dental plaque, the protective effects of fluoride on teeth and causes of tooth decay. A significantly higher number of immigrants also reported that a dentist had shown them a tooth-brushing technique and were proficient in English. More than one-third of refugee participants had never been to a dentist for any preventive or restorative services.

## Discussion

To our knowledge, this study is the first to document the oral health status and needs of this population in Saskatchewan, Canada. The mean dmft/DMFT scores were 5.80 ± 4.24 and 3.52 ± 3.78 for the refugees and the immigrants respectively, which is considerably higher than the scores of children (0.49), aged 6–11 years born in Canada [21]. Dental caries was found in 15.1 % of the immigrant teenagers, but only in 3.8 % of children born in Canada [22]. Further, a study conducted in Nova Scotia, Canada reported a large proportion of Bhutanese immigrants and refugees had untreated caries and gingivitis [23].

Data from the Saskatchewan Dental Health Screening Program 2008-09 suggest that among 8835 grade 7 students (age 12) living in the province, the average DMFT was only 0.80 [24]. Within the same age group, 11.4 % of these students did not need dental treatment, while 66.2 % of them were caries free in their permanent dentition. Similar results were reported in a much younger cohort of 9079 grade 1 students aged 6 (dmft/DMFT was 3.14). The Saskatchewan Dental Health Screening Program, 2008–2009 report also indicated that 41.5 % of the same age group of grade 1 students were caries-free, and 27.1 % of them did not require any restorative dental therapy.

Findings from our study and others carried out in Canada suggest that the dmft/DMFT scores for recent immigrants and refugees are higher than the national norm and hence do not meet the Canadian Oral Health Strategy Guidelines (2010) for children in grade one. Maserejian et al. [25] compared the caries experience of children of immigrants to the children of US-born caregivers at enrollment and new caries increments during the 5-year New England Children’s Amalgam Trial. As expected, at baseline, the children of immigrants had more carious lesions than residents. They also reported that Immigrant status and language preference were not associated with 5-year caries increments.

The feedback from our questionnaire revealed that, in general, immigrants and refugees have other priorities and concerns such as the stress associated with adapting to a new culture, learning English as a second language, issues with transportation, finance, and finding schools for children to deal with before they can consider their children’s oral health needs [26]. Even if they do desire to seek dental care, most find the cost of regular treatment at a private dental office, prohibitive, especially since most newcomers do not have dental insurance or the financial means to meet the cost. Similar results were reported in a group of 48 mothers of 3–5 year-old children from selected African communities in Alberta, Canada [27]. These immigrant mothers had been living in Canada for 5 years or less. Access barriers were associated with parental knowledge of preventive services, English skills, and external constraints concerned dental insurance, social support, time, and transportation. Inadequate dental coverage appears to be associated with low income as evidenced by a recent national survey with a group of Canadian adults [28]. This survey reported that only a small percentage (19.3 %) of the lowest income group had private dental insurance compared with 80.5 % of the highest income group. Such barriers may impede regular visits to a dental office and negatively impact oral health outcomes in vulnerable population groups.

### Limitations of the Study

Limitations of the study include the relatively small sample size and convenience sampling approach to data collection. Therefore, our results may not be generalizable to all immigrant and refugee children. Another limitation is that we had a few families with more than one child enrolled in the study. This may have created biases in our results. However, since all families were similar in terms of their poor socioeconomic status, we do not expect a high level of bias in our results.

## Conclusion

According to our study, refugee children have higher dmft/DMFT scores than immigrant children; both of these groups are at a greater risk when compared to their native counterparts and this finding is consistent with studies done elsewhere [18]. These results can be used as a basis for more comprehensive assessments intended to plan publicly-funded oral health programs specifically targeting recent immigrants and refugees in the province of Saskatchewan. Focussed interventions can reduce the burden of disease on these populations and on funding agencies as well.
